# Ageist Communication Experienced by Middle-Aged and Older Canadians

**DOI:** 10.3390/ijerph19042004

**Published:** 2022-02-11

**Authors:** Alison L. Chasteen, Sali A. Tagliamonte, Katharina Pabst, Samantha Brunet

**Affiliations:** 1 Department of Psychology, University of Toronto, Toronto, ON M5S 3G3, Canada; samrbrunet@gmail.com; 2 Department of Linguistics, University of Toronto, Toronto, ON M5S 3G3, Canada; sali.tagliamonte@utoronto.ca (S.A.T.); katharina.pabst@mail.utoronto.ca (K.P.)

**Keywords:** ageism, age stereotypes, experiences

## Abstract

Ageism has been well-documented in the United States, but ageism experiences in Canada remain less well-known. To address this gap, in the current research middle-aged and older Canadians completed a conversational interview in which they described their ageism experiences. Their descriptions were coded for life domain, perpetrator, and type of ageist communication. The most common domain in which ageist communication occurred was the public sphere, with perpetrators most often being strangers. Ageist communication most often involved age-based social or physical assumptions about the participant. In combination, these findings detail how ageism manifests in the everyday lives of Canadians and contribute to understanding the nuances of the expression of ageism in North America.

## 1. Introduction

In 2021, the World Health Organization released a global report on ageism [1]. Although the original definition of ageism focused on prejudice between age groups [2], the WHO expanded this definition to include age-based stereotypes (i.e., beliefs), age-based prejudices (i.e., feelings), and age-based discrimination (i.e., behaviors) [1]. The WHO report reflects the large and growing area of research showing the prevalence of ageism worldwide, as manifested in negative attitudes toward older adults (e.g., [3,4,5,6,7,8,9]) and older people’s own reported experiences of being the targets of ageist comments and behavior (e.g., [10,11]). A recent systematic review detailed the numerous harms arising from ageism, including worsened mental, physical, and cognitive health; poor quality of life; lower well-being; poor social relationships and social isolation; and reduced longevity [12]. Given the negative impacts of ageism on older people, it is vital to understand how ageism is experienced in order to develop interventions to counteract it. 

A recent example of how ageism is experienced came from a national poll of American older adults, which found that 82% experienced one or more forms of ageism (ageist messages in media, internalized ageism, and ageism in interpersonal interactions) in their daily lives [13]. Of those reporting ageism experiences, 45% encountered ageism in interpersonal interactions, which took the form of age-based assumptions about their physical, sensory, and cognitive abilities as well as beliefs that they needed assistance in tasks they could complete independently [13]. 

Similar themes emerged in a study by Chasteen et al. [14], which provided a relatively detailed picture of ageism experienced by a mostly American sample of young, middle-aged, and older adults. Participants’ written descriptions of an ageism incidents were coded for the life domain (e.g., work, public space), type of perpetrator (e.g., employer/co-worker, stranger), and type of act (e.g., unwanted help, lack of respect). They found that older adults experienced ageism when receiving goods and services, at work, and in public spaces, whereas younger age groups reported experiencing ageism most often at work. Consistent with that pattern, the perpetrators of ageist acts tended to match the domain in which the prejudiced behavior occurred (e.g., by a stranger in a public space). In terms of different types of ageist acts, older adults encountered incorrect assumptions about themselves, unwanted help, and a lack of respect, and this differed from the younger age groups, who also reported experiencing some incorrect assumptions and a lack of respect, but no unwanted help.

Although the findings of Chasteen et al. [14] provide a more nuanced understanding of the shape and form that ageism experiences can take, more research is needed to determine whether the patterns observed can be generalized to other older adults. Moreover, research on ageist attitudes towards older people as well as on older adults’ own experiences of ageism tends to rely on surveys or polls to capture individuals’ experiences. The present study aimed to address these issues by using informal interviewing techniques developed in sociolinguistics [15] in a sample of middle-aged and older Canadians in order to determine whether similar patterns of ageism experiences would be found when individuals had great latitude to describe an ageism incident. In doing so, this study provides insight into ageism both in the context of extended story-telling and in a less-studied North American population—namely, Canadians. As indicated in the Chang et al. review, the number of studies on ageism from the United States far outpaces those from other nations [12]. Broadening the scope of ageism research is needed in order to develop tailored interventions. Moreover, it has been suggested that ageism experiences may differ between Americans and Canadians [16]. Thus, the present study represents a conceptual replication designed to ascertain the degree to which ageism experiences assessed previously in one country (United States) using a survey method can also be observed in another country (Canada) using an interview method. We note that this study was conducted prior to the onset of the COVID-19 pandemic, which has been shown to have exacerbated ageism [17,18,19,20]. Although it is important to capture acts of bias such as ageism during a time of crisis, we believe it is also vital to ascertain the shape and form of age prejudice during less extraordinary times.

Based on the earlier findings of Chasteen et al. [14], we anticipated the following results. Middle-aged adults would report experiencing ageism most often at work, whereas older adults would indicate goods and services as the most common domain. Perpetrators would match the domain in terms of frequency (e.g., at work by an employer/co-worker). As for the type of ageist act, we anticipated that both age groups would report experiencing incorrect assumptions and a lack of respect, but that only older adults would indicate receiving unwanted help. 

## 2. Materials and Methods

### 2.1. Participants

A total of 88 adults participated in the study. The sample comprised 32 middle-aged adults (aged 47–59, *M* = 53.94; *SD* = 3.29; 67.7% female; 89.7% White; 71.0% Bachelor’s Degree or higher), and 56 older adults (aged 60–90, *M* = 72.57, *SD* = 9.02; 50.9% female; 93.8% White; 50.8% Bachelor’s Degree or higher). The qualitative data collected for this study were part of a larger project on language variation and aging that involved extended conversational interactions with participants in their homes. Thus, the sample size was determined by the needs and feasibility of the larger project. 

During 2018–2019, community-dwelling middle-aged (defined as 35–59) and older (defined as 60+) adults were recruited from the Toronto English Archive project in the Department of Linguistics, from the Adult Volunteer Pool in the Psychology Department, as well as from the Greater Toronto Area through advertising and flyers. Eligibility criteria for the study were determined by the needs of the larger sociolinguistic project and included the first language being English and having been born and raised in the Greater Toronto Area. The scheduling of conversational interviews occurred at participants’ convenience, typically 1 to 3 weeks after a recruitment phone call and eligibility check. This study was approved by the Research Ethics Board of the University of Toronto. 

### 2.2. Procedure

The data for the current study come from what are referred to in sociolinguistics as “sociolinguistic interviews”, a series of hierarchically structured questions designed to elicit narratives of personal experience over one or two hours of face-to-face conversation with a single participant and an interviewer [15,21,22]. Before the interviews began, the participants completed an informed consent form. Towards the end of each interview, following the open-ended questions that were part of the sociolinguistic modules of the interview, participants were asked to describe an event in which they were treated differently because of their age and indicate the person who perpetrated the ageist behavior. As part of a separate experimental testing session in a social psychology laboratory at the university, participants completed a demographic questionnaire. At the end of the second session, participants were debriefed and compensated $60 CAD.

#### Coding Procedure

A team of research assistants transcribed the recorded interviews using orthographic methods and a protocol designed for consistency and efficient data exploration [23]. The section of the interview pertaining to participants’ experiences of ageism was then reviewed by two independent raters who first determined whether participants had described an experience of ageism, and if so, whether that incident was aimed at themselves or someone else. 

The descriptions of an ageism incident experienced directly by the self were then assessed using a coding scheme developed in prior work [14]. The coding scheme includes: life domain categorized into six types (work, family, social, education/school, goods and services, public space); perpetrator relationship to the target (family member, friend, stranger, service worker, employer/co-worker, authority figure); and type of ageist act, categorized into five types (social exclusion, cognitive assumptions, lack of respect, social/physical assumptions, unwanted help/special treatment). See Supplementary Materials for more details about the coding scheme. 

Although a single domain and perpetrator were identified for each ageism incident, some incidents were characterized by multiple types of ageist acts. When responses could not be classified into one of these categories, they were deemed “not codable” and were excluded from the analysis. In addition, the coders also rated each description for valence using a 5-point scale that ranged from (1) very negative to (5) very positive. 

## 3. Results

### 3.1. Analytic Approach

Since the participants were asked to describe an ageism event that they experienced themselves, we excluded responses that did not describe an ageism event (*n* = 20) and that were not aimed at the participants themselves (*n* = 10). Frequency percentages of the remaining sample (*n* = 58; 21 middle-aged, 37 older adults) were then assessed via Chi-square tests of independence, unless otherwise noted.

### 3.2. Valence of Ageism Experiences

An independent samples t-test showed no difference in the valence of middle-aged (*M* = 2.67, *SD* = 1.11) and older adults’ (*M* = 2.92, *SD* = 1.48) descriptions of their experiences of ageism, *t*(56) = −0.679, *p* = 0.50. Both age groups’ descriptions on average were somewhat negative to neutral in valence. Below is an example of an experience from an individual, 049, (age 71) that was rated as very negative. Note that for all examples, numbers in square brackets are unique numerals assigned to each participant. Excerpts have been edited slightly for readability:

[049] Oh yeah. Yeah. I get called ‘dearie’. *Interviewer:* Really? [049] ‘Honey’ you know. You know salespeople. *Interviewer:* Oh. [049] “Oh dear- oh sweetie can I help you? Dearie, can I help you?” And I’ll go, “No darling, you can’t.” (both laugh) And they look at me and I think- at one point I went and I said- I called a manager and said, “You have to get your staff to stop calling older people ‘dearie’ and ‘sweetie’. We are not their dearie and we are not their sweetie.” *Interviewer:* Yeah. [049] “We’re not their grandmother.” *Interviewer:* Yeah. [049] And they looked at me like, “So sorry.” or ‘Hon’. “Hon, can I help you?” “No Hon, you can’t.”

### 3.3. Domain of Ageism Experience

There was a remarkable similarity between the two age groups in terms of the reported domain of the ageist experience (*X*^2^ (4, *N* = 53) = 2.65, *p* = 0.62). As shown in Table 1, Panel A, middle-aged adults reported ageism most frequently in public spaces, at work, and when receiving goods and services. A similar pattern occurred for older adults, with an even greater percentage reporting ageism most frequently in public spaces, followed by at work and then in goods and services settings. 

### 3.4. Perpetrators of Ageist Act

Mirroring the pattern for domain, middle-aged and older adults reported similar perpetrators of ageism (*X*^2^ (5, *N* = 57) = 6.22, *p* = 0.29). Table 1, Panel B shows that middle-aged adults most often reported ageism perpetrated by strangers, followed by employer/co-workers and service workers. This pattern was also observed in older adults’ reports, whereby an even greater percentage indicated ageism perpetrated by strangers, followed by employer/co-workers and then a smaller percentage of service workers. The two age groups differed regarding reports of friends and authority figures as perpetrators. Middle-aged adults indicated more instances of friends as perpetrators, whereas older adults indicated more instances perpetrated by authority figures (i.e., someone such as a teacher, coach, doctor, or another person from whom the target takes and obeys instruction). 

### 3.5. Type of Ageist Experience

Middle-aged and older adults also experienced similar types of ageist actions (*X^2^* (4, *N* = 54) = 3.25, *p =* 0.52). Table 1, Panel C shows that middle-aged adults reported experiencing social/physical assumptions most frequently, followed by a lack of respect and unwanted help/special treatment. Older adults also reported experiencing social/physical assumptions the most, followed by a lack of respect. Whereas those two experiences constituted the majority of middle-aged responses, there was greater diversity in older adults’ experiences, with some older participants also reporting instances of unwanted help/special treatment, social exclusion, and cognitive assumptions. An example of a middle-aged adult’s, [064, age 56], report of experiencing ageism in a public space in the form of social/physical assumptions and unwanted help/special treatment is:

When I’m on the bus there are some people now offering me their seats. And I kind of- sometimes I’m kind of taken aback by that like what are you asking me for? I’m- I’m not that old. I’m- I’m- do I look it, you know? But then I appreciate it and I say thank you but you know it- it kind of also on the other side makes me feel old.

Whereas, for an older adult [026, age 73], an example is:

I think, when you get old, on the street you become invisible. Because it seems like everybody who is not old, wants to bang into you because they don’t see you. Well, there’s a lot of this going on, too. But it- you know, like I just thought- like, back in- way back in our day, you know, you would step aside for an older person and that kind of- but now, that’s the only thing I notice. Other than that, I don’t think anybody treats you any differently. Um, I do get offered a seat on the subway, to which I scowl at them.

## 4. Discussion

The goal of the present study was to examine ageism experiences described by middle-aged and older adults. We used a conversational interview method to allow for the greater expansion of people’s experiences. In addition, we captured ageism experiences among Canadian individuals, an understudied group. Thus, the current work provides a conceptual replication by using different forms of assessment in a less investigated population. 

Unlike in previous findings [14], there were no age differences in the shape or form in which ageism experiences occurred. Middle-aged and older participants showed similar patterns for the domain in which the incident occurred, the perpetrator of the ageist act, and for the type of ageist act. In the case of the domain, both age groups most often recalled ageism incidents that occurred in public spaces. This contrasts with earlier findings [14], in which middle-aged adults most often reported ageism experiences in the workplace and older adults most often reported experiences when receiving goods and services. As in our earlier study, the perpetrators of ageism largely mapped onto the domain in which the incident occurred. In the present study, this meant strangers were reported as the most common transgressor, consistent with recollections of ageist acts in public spaces. Notably, strangers expressing ageism in public spaces are considered to be among the least acceptable sources of ageism [24]. It appears that such normative beliefs, however, are not a strong enough deterrent to strangers engaging in ageist communication and acts in the public sphere. 

In terms of types of ageist acts, social/physical assumptions were reported by both middle-aged and older adults far more than any other type of act, followed by a lack of respect. Although these two forms of ageist behavior were the most common in Chasteen et al. [14], other forms of ageist behavior were also recalled, whereas in the present study very few or no participants reported social exclusion or cognitive assumptions. Lack of respect was experienced more by both age groups in this study compared to past findings. Both groups also reported experiencing some unwanted help, although that was less frequent than in the earlier study. When it did occur, unwanted help sometimes took the form of offering seats on public transportation. While that might appear to be a courteous act, some participants viewed such offers as a questioning of their strength, vitality, and independence, thus making them feel older. This suggests that, when offering help, it is important to consider the recipient’s perspective. 

There are some potential reasons why we did not observe age differences in the present study. First, the middle-aged adults in this sample were older on average (*M* age = 53.94 years) compared with those in the study of Chasteen et al. [14] (*M* age = 42.15 years). It could be that the middle-aged participants in the current study appeared somewhat older and thus encountered more ageist behavior in public spaces. It should be noted, however, that the vast majority (93.5%) of the middle-aged participants were still working, so it is somewhat surprising that the workplace was not mentioned more frequently as a domain in which ageism was occurring. Second, the sample in the present study was smaller than that examined by Chasteen et al. [14]. This was due to the fact that these data were part of a larger sociolinguistic and social psychological study that included the interactive and extensive interviewing format of sociolinguistics; thus, the sample size was determined by the overall project. Third, it may be that because the prompt regarding ageism experiences was at the end of the interview, following a lengthy conversation on other topics, the participants focused on different ageism experiences than they would have when completing a survey that directly and solely inquired about ageism encounters (e.g., Chasteen et al. [14]).

Overall, the present data provide valuable insight into the ageism experiences of middle-aged and older Canadians. Moreover, parallel findings to earlier research were obtained, despite the fact that the ageism comments emerged out of stories of personal experience rather than from self-report surveys. As such, this study provides a necessary extension of past findings to an understudied population using a different method. 

Although the contributions of this work are notable, there are limitations to this study. The constraints of the smaller sample size were likely a factor in the lack of age differences, both in terms of statistical power and also in terms of the age range. Indeed, there were only nine participants aged 80+, and although their reported ageism experiences were similar to those aged 60–79, a larger sample would be needed for statistical comparisons. Given the heterogeneity among older adults [17,20], it is conceivable that comparisons between younger and older segments of the older adult age group might reveal differences. In order to conduct a conceptual replication, we used the same prompt as in our earlier work in which we asked participants to report instances in which they were treated differently because of their age. Future studies may wish to use more specific queries about experiences of age-based prejudice. Additional directions for future research include considering how possible individual differences (e.g., moral or political views, need for care or assistance) might moderate ageism experiences. 

## 5. Conclusions

Combatting ageism requires a deep understanding of how it manifests in older adults’ everyday lives. The present study undertook that challenge and demonstrated that ageism experiences are varied and can occur in a variety of life domains, be committed by different perpetrators, and be expressed in different ways. By understanding the nuances of ageism expression, we can develop better tools to reduce age biases and improve intergenerational relations, an important goal whether during ordinary or extraordinary times.

## Figures and Tables

**Table 1:** Comparing middle-aged and older adults’ responses for (A) ageism domain, (B) perpetrator, and (C) experience type.

	Middle-Aged Adults (%)	Older Adults (%)
(A) Ageism Domain		
Work	20.0	15.2
Social	5.0	0.0
Family	5.0	6.1
Education/School	0.0	0.0
Goods and Services	15.0	9.1
Public Space	55.0	69.7
(B) Perpetrator		
Family Member	5.0	8.1
Friend	10.0	0.0
Stranger	55.0	62.2
Service Worker	15.0	8.1
Employee/Co-Worker	15.0	13.5
Authority Figure	0.0	8.1
(C) Experience Type		
Social Exclusions	0.0	5.6
Cognitive Assumptions	0.0	8.3
Lack of Respect	22.2	13.9
Social/Physical Assumptions	72.2	63.9
Unwanted Help/Special Treatment	5.6	8.3

*Note.* There were no significant differences by age group (all ps > 0.29).

## Data Availability

Considering this study uses narrative audio data, only non-identifiable data may be shared with other researchers for purposes of reproducibility.

## Supplementary Materials

The following supporting information can be downloaded at: https://www.mdpi.com/article/10.3390/ijerph19042004/s1, Coding Scheme Used to Identify Domains, Perpetrators, and Types of Ageism Experiences
