# Supplementary material for: Ageist Communication Experienced by Middle-Aged and Older Canadians
Source: Int J Environ Res Public Health. 2022 Feb 11;19(4):2004. doi: 10.3390/ijerph19042004 (PMC8871782; doi:10.3390/ijerph19042004)
Supplement: Supplementary file 1 [file ijerph-19-02004-s001.zip › ijerph-1528792-supplementary.pdf]

Supplementary Material

Acknowledgement: Re-printed with permission of Oxford University Press. The material below originally appeared in: Chasteen, A. L.; Horhota, M.; Crumley-Branyon, J. J. Overlooked and underestimated: Experiences of ageism in young, middle-aged, and older adults. *The Journals of Gerontology, Series B: Psychological Sciences* **2021**, *76*, 1323–1328, https://doi.org/10.1093/geronb/gbaa043.

Coding Scheme Used to Identify Domains, Perpetrators, and Types of Ageism Experiences:

<u>Domain</u>

1. Work: the incident or series of incidents occurred in the workplace
2. Social: in a situation with friends or acquaintances, such as a party or social outing
3. Family: situations in which the person is being impacted by their families, through general interactions or more specific events
4. Education/ School: Interactions with other students, faculty or staff within a school or a school related event
5. Goods & Services/ Shopping: Interactions such as those that might take place in a grocery store or shopping mall with fellow shoppers or service workers
6. Public Space: interactions with strangers in places that are not considered private, such as a conversation taking place at a bus stop
7. Not codeable: interactions that do no align with any of the aforementioned categories

<u>Perpetrator</u>

1. Family member: the target's interaction took place with a family member
2. Friend: interaction took place with a person who is an acquaintance
3. Stranger: interaction took place with a person who is unfamiliar to the target
4. Service worker: interaction took place with someone such as a cashier or postal worker
5. Employer/co-worker: interaction took place with someone that the target works for/with
6. Authority figure: interaction took place with a person such as a teacher, coach, or other person from whom the target takes and obeys instruction
7. Not codeable

<u>Experience type</u>

1. Social Exclusion: could include being left out of groups due to age limit laws or interactions such as ostracizing a person for not being 'hip' or not being able to keep up with the times
2. Cognitive Assumptions: assuming, based on presumptive age, that a person doesn't have the competence, knowledge, or experience to fulfill some duty or take a job

3. Lack of Respect: actively expressing lack of interest in a person's opinions or actions based on age; dismissive invalidations.
4. Social/Physical Assumptions: assuming that a person is older or younger than they are, or making assumptions based on age, such as assumptions of values, opinions, or behaviors
5. Unwanted Help/Special Treatment: benevolent acts such as offering unprompted help or giving special treatment
6. None: the target has experienced no prejudice based on age
7. Not codeable: interactions that do not align with any of the aforementioned categories
